# Characterization and determination of six flavonoids in the ethnomedicine “Dragon’s Blood” by UPLC-PAD-MS

**DOI:** 10.1186/1752-153X-6-116

**Published:** 2012-10-10

**Authors:** Tao Yi, Yina Tang, Jianye Zhang, Zhongzhen Zhao, Zhijun Yang, Hubiao Chen

**Affiliations:** 1 School of Chinese Medicine, Hong Kong Baptist University, Hong Kong Special Administrative Region, P. R. China

**Keywords:** Dragon's Blood, *Daemonorops draco*, UPLC-PAD-MS, Characterization, Quality evaluation

## Abstract

**Background:**

“Dragon’s Blood” (DB) has long been used as an ethnomedicine in China to invigorate blood circulation for the treatment of traumatic injuries, blood stasis and pain. To comprehensively assess the quality of DB medicine, a precise and accurate method that can rapidly separate, characterize and quantify multiple active components of DB is crucial.

**Results:**

An ultra performance liquid chromatography (UPLC) coupled with photodiode array detection (PAD) and electrospray ionization mass spectrometry (ESI-MS) method was developed for characterization and determination of six flavonoids in DB. A comprehensive validation of the developed method was conducted, and confirmed that the method presented good sensitivity, precision and accuracy. All linear regressions were acquired with *R*^2^ > 0.99, and the limits of detection ranged from 0.06 to 0.83 ng. The relative standard deviation (RSD) values were found to be within the range 1.4–3.8% for the method repeatability test. Recovery studies for the quantified compounds were found to be within the range 94.2–102.8% with RSD less than 4.9%. DB samples collected from different geographical regions were analyzed by the present method, and the results demonstrated that the contents of the six flavonoids in DB samples varied significantly. Three major active components among the six flavonoids, namely dracorhodin, (2S)-5-methoxyflavan-7-ol and (2S)-5-methoxy-6-methylflavan-7-ol, are suggested as the index for DB quality evaluation.

**Conclusions:**

Overall, the present hyphenation method is highly efficient and reliable, and hence suitable for the characterization and determination of the flavonoids of DB ethnomedicine.

## Background

“Dragon’s Blood” (DB), a deep red resin secreted from the fruit of the *Daemonorops draco* tree, has long been used as an ethnomedicine in China to invigorate blood circulation for the treatment of traumatic injuries, blood stasis and pain [1,2]. Flavonoids [3,4] and resin terpenoid acids [5,6] are the main constituents of DB. Currently, the quality evaluation for DB medicine is based on the content of only one marker compound, namely dracorhodin, which is one of the bioactive compounds identified so far in DB [1].

In recent years, pharmacologic studies have demonstrated that DB medicine exerts its clinical effects by inhibiting blood platelet aggregation [2,7], and more components of DB have been found to be active in this process. For example, it is reported that the raw extract of DB dose-dependently inhibits myocardial ischemia and thrombus formation of the rats, and the coagulation time is extended significantly [8]. (2S)-5-methoxy-6-methylflavan-7-ol, a flavonoid isolated from DB, dose-dependently inhibits aggregation of washed rabbit platelets induced by collagen, arachidonic acid and adenosine diphosphate (ADP) [9].

It is widely known that multiple constituents are probably involved in any herb's therapeutic functions, and that the content of a single marker compound cannot accurately reflect the quality of herbal products [10,11]. Thus, for DB as an herbal medicine, a novel quality evaluation standard based on the contents of multiple active components is needed to comprehensively assess its quality. However, accurate means for qualitative and quantitative analysis of the multiple components simultaneously have not been reported, even though the determination of dracorhodin in DB has been carried out by TLC [12], HPLC [13] and CE [14], respectively. Therefore, a method for the simultaneous characterization and determination of the major active components in herbal medicines in general and DB in particular is still a top priority for accurate quality evaluation.

In the present study, a method coupling ultra-performance liquid chromatography (UPLC) with photodiode array detection (PAD) and electrospray ionization mass spectrometry (ESI-MS) was developed for the characterization and determination of six flavonoids in DB medicine. The validation results revealed that the developed method is highly efficient and reliable, and hence suitable for qualitative and quantitative analysis of DB samples. Based on the sample assay results, three major active components among the six flavonoids, namely dracorhodin, (2S)-5-methoxyflavan-7-ol and (2S)-5-methoxy-6- methylflavan-7-ol, are suggested as the index for quality evaluation of DB medicine.

## Experimental

### Materials

“Dragon’s Blood” (DB) samples were collected from various regions of China. Identity of the samples was confirmed by the authors, and voucher specimens were deposited in the School of Chinese Medicine, Hong Kong Baptist University.

### Reagents and chemicals

Analytical grade methanol (Labscan, Bangkok, Thailand) was used for preparation of standards and sample extraction. Chromatographic grade acetonitrile (Labscan, Bangkok, Thailand), chromatographic grade formic acid (Fluka, Buchs, Switzerland) and deionized water obtained from a Milli-Q water purification system (Millipore, Bedford, MA, USA) were used for preparation of the mobile phase.

**Figure 1:**
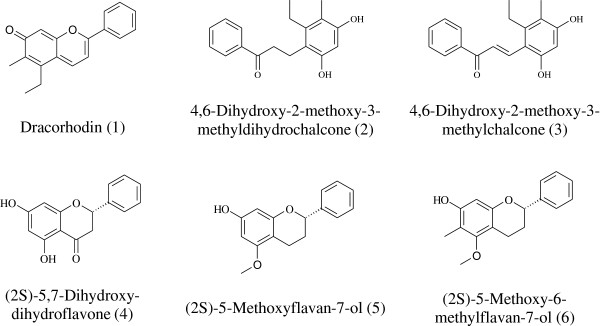
Chemical structures of the identified components in the UPLC chromatograms.

The standard compound of dracorhodin perchlorate was purchased from the National Institutes for Food and Drug Control (Beijing, China). 4, 6-dihydroxy-2-methoxy-3-methyldihydrochalcone, 4, 6- dihydroxy-2-methoxyv-3-methylchalcone, (2S)-5,7-dihydroxy-dihydroflavone, (2S)-5-methoxyflavan- 7-ol and (2S)-5-methoxy-6-methylflavan-7-ol were isolated by our laboratory with a purity of more than 98%, and their chemical structures were elucidated by comparing them with published data of ^1^H and ^13^C NMR [15-17]. Details of their separation and structural elucidation will be reported elsewhere. Their chemical structures are shown in Figure 1.

### Preparation of standard and sample solutions

The stock solutions of six standard compounds were prepared in methanol and stored in the refrigerator. The working solutions were prepared by appropriate dilution of the stock solutions with methanol, and the resulting concentration ranges are listed in Table 1.

**Table 1:** **Linearity curves**, **LOD and LOQ for six compounds**

**Peak**	**Compounds**	**Linear equations**	**Range(μg/mL)**	***R***^**2**^	**LOD(ng)**	**LOQ(ng)**
1	Dracorhodin	y = 8580.5x + 8536.8	5-250	0.9998	0.48	1.60
2	4,6-Dihydroxy-2-methoxy-3- methyldihydrochalcone	y = 6472.4x + 1430.2	1-50	0.9982	0.15	0.50
3	4,6-Dihydroxy-2-methoxy-3- methylchalcone	y = 6529.5x - 2240.9	1-50	0.9999	0.52	1.73
4	(2S)-5,7-Dihydroxy- dihydroflavone	y = 25710.1x + 14943.4	1-50	0.9989	0.06	0.20
5	(2S)-5-Methoxyflavan-7-ol	y = 989.1x + 16685.5	20-1000	0.9992	0.83	2.76
6	(2S)-5-Methoxy-6-methyl- flavan-7-ol	y = 4098.2x + 14161.0	5-250	0.9991	0.21	0.70

DB sample powder (0.1 g) was extracted with 10 mL of methanol by means of sonication at room temperature for 0.5 h. Sampling weight of DB is adjusted if necessary. The operations were repeated once, and the residue was washed with 4 mL of methanol. Total extracts were combined in a 25-mL volumetric flask, which was filled up to the calibration mark with extraction solvent. The extracts were then filtered through a syringe filter (0.2 μm, Alltech, Beerfield, IL, USA). An aliquot of 2 μL solution was injected for UPLC-PAD-MS analysis.

### UPLC-PAD-MS conditions

A Waters Acquity^TM^ ultra performance liquid chromatography (UPLC) system (Waters Corp., Milford, USA) with photodiode array detection (PAD), was hyphenated to a Bruker MicrOTOFQ system by an electrospray ionization (ESI) interface (Bruker Daltonics, Bremen, Germany) for chromatographic and mass spectrometric (MS) analysis. Data analysis was conducted using DataAnalysis software version 4.0 (Bruker Daltonics). For chromatographic separation, a Waters BEH C_18_ column (1.7 μm, 2.1 × 100 mm) with a VanGuard^TM^ pre-column (BEH, C_18_, 1.7 μm, 2.1 × 5 mm) was used. The mobile phase consisted of 0.1% formic acid in water (A) and 0.1% formic acid in acetonitrile (B) using a gradient program of 25% (B) in 0–2 min and 25-55% (B) in 2–15 min. The solvent flow rate was 0.3 mL/min, the column temperature was set to 40°C, and the detection wavelength was 280 nm. The conditions of MS analysis in the positive ion mode were as follows: drying gas (nitrogen), flow rate, 8 L/min; gas temperature, 180°C; scan range, 50–1600 m/z; end plate offset voltage, -500 V; capillary voltage, 4500 V; nebulizer pressure, 2.5 Bar.

### Method validation and sample analysis

The calibration curve was established by plotting the peak area against the concentrations of the standards with linear regression analysis. The detection (LOD) and limit of quantitation (LOQ) of the quantified constituents were visually evaluated with a signal-to-noise ratio of about 3:1 and 10:1, respectively. Instrumental precision was investigated by repeatedly analyzing the same mixed standard solution six times, and the method repeatability was evaluated by six replicated analyses of the same DB sample. Recovery of all the quantified constituents was determined by samples at different concentration levels using a mixture of standards with 50, 100 and 200% of the quantified levels of constituents in the DB sample. All DB samples collected from various regions were analyzed using this method.

## Results and discussion

### Optimization of the extraction method and analysis conditions

Compared to the reflux and soxhlet extraction, sonication extraction was easier, while still satisfactory. The extraction solvent was chosen from methanol, ethanol and their various concentrations of aqueous solution. The results revealed that extraction with absolute methanol produced the highest yield for the desired analytes. Thus, methanol was chosen as the solvent for the sample extraction. Extraction times and cycles were further optimized, and the results demonstrated that exhausted extraction could be achieved when DB sample powder of 0.1 g was extracted with 10 mL of methanol by means of sonication for 0.5 h, twice.

**Figure 2:**
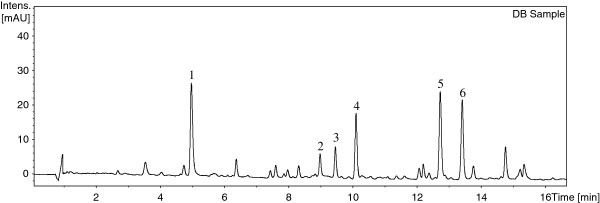
Typical UPLC chromatogram of DB sample using PDA at 280 nm.

The conditions for chromatographic analysis including mobile phase composition, type of column, column temperature and detection wavelength, were optimized based on our previous study [18]. Acetonitrile was found to be preferred over methanol as the mobile phase because it gave better separation for the analytes at a lower column pressure. Mobile phase gradients were compared on a HSS C_18_ column and BEH C_18_ column at different temperatures. The results showed that satisfactory separation could best be obtained by eluting DB samples on a BEH C_18_ column at 40°C using a linear gradient of acetonitrile and water within 15 min. A wavelength of 280 nm was chosen to monitor the analytes after comparing the chromatograms of the DB samples recorded at wavelengths within 190–500 nm. A typical UPLC chromatogram of DB sample at 280 nm is shown in Figure 2.

The mass spectrometric conditions were optimized in both positive and negative ion modes; the positive ion mode was found to be more sensitive. In order to promote the formation of quasi-molecular ions [*M*+H] ^+^ in MS analysis, 0.1% formic acid was used in the mobile phase. MS offset voltage was further adjusted to generate characteristic fragments of the six analytes.

### ESI-MS characterization of the six flavonoids

Herbal medicine is a multicomponent system with many unknown components, and the chromatographic peaks often overlap when a sample of an herbal medicine is eluted on a column. The retention time of the analytes can vary between different laboratories, while the UV spectra of the active components and other analogues are greatly similar. Thus, the direct identification of the target compounds merely based on the chromatographic parameters is still a challenge in many cases.

Compared to chromatography, mass spectrometry can provide more exclusive molecular information on the target compounds by elucidating their fragmentation pathways. These MS fragmentation characteristics greatly contribute to identify the active components from the complex mixture of herbal medicine even if the standard compounds are unavailable. Therefore, precise MS characterization of the six flavonoids is necessary in this study.

**Figure 3:**
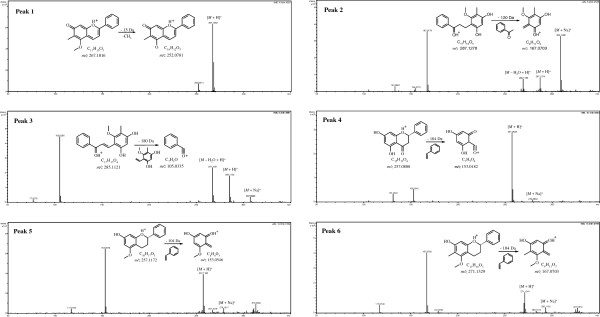
**EIC**-**MS spectra and proposed fragmentation pathways of the six components.**

Based on comparison with chromatograms and MS fragments of standard compounds, six peaks in the DB sample chromatogram were unambiguously identified as dracorhodin (1), 4,6-dihydroxy-2- methoxy-3-methyldihydrochalcone (2), 4,6-dihydroxy-2-methoxy-3-methylchalcone (3), (2S)-5,7- dihydroxy-dihydroflavone (4), (2S)-5-methoxyflavan-7-ol (5) and (2S)-5-methoxy-6-methylflavan- 7-ol (6). From the results, it is obvious that the major types of constituents in DB are flavonoids, including anthocyanins, chalcones, flavanones and flavans. The extracted ion chromatogram (EIC) MS spectra and the proposed fragmentation pathways of the six flavonoids are shown in Figure 3.

Under the MS conditions, dracorhodin (peak 1) is more stable than other components, and it is not easy for dracorhodin to generate new characteristic fragment ions. Chalcones (peak 2 and peak 3) lose a common molecule of H_2_O (−18 Da) forming their *M* + H] ^+^ ions, while the fragments of m/z 167 (C_9_H_11_O_3_) and m/z 105 (C_7_H_5_O) are the characteristic ions of peak 2 and peak 3, respectively. Flavanone (peak 4) and flavans (peaks 5 and 6) lose a common molecule of C_8_H_8_ (−104 Da) to generate their characteristic fragment ions of m/z 153 (C_7_H_5_O_4_), m/z 153 (C_8_H_9_O_3_) and m/z 167 (C_9_H_11_O_3_), respectively [17]. The detailed elemental composition of the fragments is summarized in Table 2, and the mass errors between the observed and calculated values of molecular weight of the fragments are found to be less than 10 mDa. The results demonstrate that the developed UPLC-MS method is specific and precise, and therefore is suitable for the characterization of the flavonoids in DB medicine.

**Table 2:** Elemental composition of major fragments in the MS spectra

**Peak**	**RT** (**min**)	**Compounds**	**Formula**	**Calculated (m/z)**	**Observed (m/z)**	**Error (mDa)**
1	4.9	Dracorhodin	C_17_H_15_O_3_	267.1016	267.1083	6.7
			C_16_H_12_O_3_	252.0781	252.0814	3.3
2	8.9	4,6-Dihydroxy-2-methoxy- 3-methyldihydrochalcone	C_17_H_18_O_4_Na	309.1097	309.1098	0.1
			C_17_H_19_O_4_	287.1278	287.1274	−0.4
			C_17_H_17_O_3_	269.1172	269.1186	1.4
			C_9_H_11_O_3_	167.0703	167.0779	7.6
3	9.4	4,6-Dihydroxy-2-methoxy- 3-methylchalcone	C_17_H_16_O_4_Na	307.0941	307.0940	−0.1
			C_17_H_17_O_4_	285.1121	285.1133	1.2
			C_17_H_15_O_3_	267.1016	267.1051	3.5
			C_7_H_5_O	105.0335	105.0385	5.0
4	10.1	(2S)-5,7-Dihydroxy- dihydroflavone	C_15_H_12_O_4_Na	279.0628	279.0630	0.2
			C_15_H_13_O_4_	257.0808	257.0838	3.0
			C_7_H_5_O_4_	153.0182	153.0242	6.0
5	12.8	(2S)-5-Methoxyflavan-7-ol	C_16_H_16_O_3_Na	279.0992	279.1017	2.5
			C_16_H_17_O_3_	257.1172	257.1188	1.6
			C_8_H_9_O_3_	153.0546	153.0616	7.0
6	13.4	(2S)-5-Methoxy-6-methyl- flavan-7-ol	C_17_H_18_O_3_Na	293.1148	293.1152	0.4
			C_17_H_19_O_3_	271.1329	271.1341	1.2
			C_9_H_11_O_3_	167.0703	167.0782	7.9

### Method validation

Validation of the developed method was conducted, and the results are summarized in Tables 1 and 3. Linearity was determined with five data points over the concentration range. For all of the analytes, good linear calibrations were achieved over the selected concentration range with *R*^2^ > 0.99. At a signal-to-noise ratio of 3:1, the limits of detection (LOD) of the quantified components were found to be within the range 0.06–0.83 ng. The instrumental precision and method repeatability were evaluated, and the relative standard deviation (RSD) values were found to be within the range of 0.8 to 2.5% for the instrumental precision test and of 1.4 to 3.8% for the method repeatability test. The accuracy of the method was validated by the determination of recovery. Recovery study was conducted using a DB sample spiked with approximately 50, 100 and 200% of known amounts of analytes in the samples. The spiked samples were then extracted and quantified in accordance with the established procedures. Triplicate sample analysis was conducted for the determination of recovery at each spiked level. The recoveries of all of the analytes were within the range 94.2–102.8% with RSD ≤ 4.9%. The results show that the overall procedure is precise and accurate, and therefore is suitable for the quantitative analysis of DB samples.

**Table 3:** Precision and accuracy study of the method

**Peak**	**Compounds**	**Precision(RSD %, *n *= 6)**		**Accuracy(RSD %, *n *= 3)**		
		**Instrument**	**Repeatability**	**Low**	**Medium**	**High**
1	Dracorhodin	1.3	1.4	102.8 (2.2)	96.2 (2.5)	98.2 (2.3)
2	4,6-Dihydroxy-2-methoxy-3- methyldihydrochalcone	0.8	2.1	94.2 (3.2)	94.7 (1.8)	95.3 (3.4)
3	4,6-Dihydroxy-2-methoxy-3- methylchalcone	1.6	3.1	99.3 (2.0)	97.7 (3.5)	100.6 (2.5)
4	(2S)-5,7-Dihydroxy- dihydroflavone	0.9	2.7	102.7 (2.8)	96.1 (1.9)	96.4 (4.9)
5	(2S)-5-Methoxyflavan-7-ol	2.5	3.8	99.9 (3.8)	101.0 (2.0)	99.8 (3.7)
6	(2S)-5-Methoxy-6- methylflavan-7-ol	1.5	2.7	101.5 (4.5)	99.8 (2.9)	98.7 (4.5)

### Sample analysis

The “Dragon’s Blood” (DB) samples collected from different regions were assessed by the present method, and the results are listed in Table 4. From the results two findings emerge.

**Table 4:** Contents of six compounds in the DB samples

**No**.	**Purchase location**	**Contents of six compounds** (**mg** /**g**, ***n*** = **3**)					
		**Dracorhodin(1)**	**4**,**6-Dihydroxy-2-methoxy-3-methyldihydrochalcone(2)**	**4**,**6-Dihydroxy-2-methoxy-3-methylchalcone(3)**	**(2S)-5,7- Dihydroxy-dihydroflavone(4)**	**(2S)-5-Methoxy flavan-7-ol(5)**	**(2S)-5-Methoxy-6-methylflavan-7-ol (6)**
DB1	Hong Kong	1.69	0.78	1.14	0.49	24.79	4.98
DB2	Hong Kong	0.93	1.11	0.46	0.47	21.01	6.60
DB3	Hong Kong	4.76	0.98	0.28	0.24	13.07	4.38
DB4	Hong Kong	0.77	0.21	0.06	0.02	4.18	3.03
DB5	Hong Kong	0.41	0.93	0.13	0.11	7.66	4.42
DB6	Beijing	15.71	10.58	4.22	2.65	87.94	23.88
DB7	Chengdu	16.46	5.69	1.36	1.21	59.32	20.01
DB8	Chengdu	8.49	2.90	1.27	0.85	42.67	11.81
DB9	Chengdu	2.64	7.85	0.26	1.44	58.78	13.70
DB10	Tianjin	30.24	17.78	1.84	3.98	167.64	47.38
DB11	Tianjin	3.04	4.63	1.96	1.30	41.78	23.48
DB12	Jilin	22.10	13.66	3.19	13.58	83.23	58.80

Firstly, there is a significant variation in the contents of the quantified components in DB samples. Such variations may be mainly due to processing of the raw material. According to the literature [19], *Daemonorops draco* is a plant indigenous to Indonesia and Malaysia, and the resin directly collected from the fruit of *Daemonorops draco* tree is called raw “Dragon’s Blood” (DB). Before being sold in markets and used in clinics, the raw DB is processed to remove fruit pulp residue, and it is shaped by the addition of excipients, such as albane and dammar. However, the cost of DB is high while the cost of the excipients is low. It is hard to tell from appearance how much true DB is in any given sample sold on the market. Hence, there is an attempt to adding more excipients into the DB, and sell it for more profit. The generally low contents of all quantified constituents in some DB samples can be attributed to this reason. On the other hand, this finding confirms the necessity of developing a comprehensive quality evaluation standard based on the quantitation of the active components and on novel analysis technology to ensure the quality of DB medicine.

Secondly, comparison of the contents of the six analytes shows that the total amounts of dracorhodin (peak 1), (2S)-5-methoxyflavan-7-ol (peak 5) and (2S)-5-methoxy-6-methylflavan-7-ol (peak 6) represent about 90% of the total quantified compounds. It is undoubted that dracorhodin applicable as markers for quality evaluation of DB, due to that it is an identified bioactive component [1]. In recent years, the anti-platelet effects of flavonoids have been confirmed by many reports in the literature [20-22], and laboratory studies suggest that “Dragon’s Blood” species exert their clinical effects by inhibiting blood platelet aggregation [2]. The anti-platelet effect of (2S)-5-methoxyflavan-7-ol and (2S)-5-methoxy-6-methylflavan-7-ol have been reported [9,23], and the underlying mechanism for anti-platelet activity of (2S)-5-methoxy-6-methylflavan-7-ol was related to inhibition of TXA2 formation via the inhibition of COX. Based on our research results and these reports, we believe that dracorhodin, (2S)-5-methoxyflavan-7-ol and (2S)-5-methoxy-6-methylflavan-7-ol can well represent the active flavonoids of DB to be responsible for the clinical effects of “Dragon’s Blood” against blood stasis.

Therefore, to reflect the roles of multiple compounds for the therapeutic functions, dracorhodin, (2S)-5-methoxyflavan-7-ol and (2S)-5-methoxy-6-methylflavan-7-ol should be chosen as analytical markers for a more comprehensive quality evaluation of DB medicine. The present hyphenation method could meet this need, making a simultaneous analysis of the three active components in a single run possible.

## Conclusions

A UPLC-PAD-MS method was developed for the simultaneous analysis of six flavonoids in the ethnomedicine “Dragon’s Blood” (DB). The six components were characterized by online ESI-MS, and then were quantified by PAD. With respect to already existing methods, the present hyphenation procedure is highly efficient and reliable, and hence suitable for qualitative and quantitative analysis of DB samples. Based on the determination results, it is suggested that three major active components among the six flavonoids, namely dracorhodin, (2S)-5-methoxyflavan-7-ol and (2S)-5-methoxy-6- methylflavan-7-ol, be used as the index for quality evaluation of DB medicine.

## Abbreviations

UPLC: Ultra performance liquid chromatography; PAD: Photodiode array detection; ESI: Electrospray ionization; MS: Mass spectrum; TLC: Thin layer chromatography; HPLC: High performance liquid chromatography; CE: Capillary electrophoresis; TXA2: Thromboxane A2; COX: Cyclooxygenase.

## Competing interests

The authors declare that they have no competing interests.

## Authors' contributions

HBC initiated and all authors designed the study. The sample extraction was conduct by YNT and JYZ. The method developments were conducted by TY who drafted the manuscript. All authors contributed to data analysis, read and approved the final manuscript.
